# Morpho-Phenological, Chemical, and Genetic Characterization of Italian Maize Landraces from the Lazio Region

**DOI:** 10.3390/plants13223249

**Published:** 2024-11-20

**Authors:** Rita Redaelli, Laura Bassolino, Carlotta Balconi, Irma Terracciano, Alessio Torri, Federica Nicoletti, Gianluca Benedetti, Valentina Iacoponi, Roberto Rea, Paola Taviani

**Affiliations:** 1 Council for Agricultural Research and Economics (CREA), Research Centre for Cereal and Industrial Crops, via Stezzano 24, 24126 Bergamo, Italy; carlotta.balconi@crea.gov.it (C.B.); alessio.torri@crea.gov.it (A.T.); 2 Council for Agricultural Research and Economics (CREA), Research Centre for Cereal and Industrial Crops, via di Corticella 133, 40128 Bologna, Italy; laura.bassolino@crea.gov.it (L.B.); irma.terracciano@crea.gov.it (I.T.); federica.nicoletti@crea.gov.it (F.N.); 3 Agenzia Regionale per lo Sviluppo e l’Innovazione dell’Agricoltura nel Lazio (ARSIAL), via Lanciani 38, 00162 Roma, Italy; gianlucabenedetti1994@gmail.com (G.B.); v.iacoponi@arsial.it (V.I.); r.rea@arsial.it (R.R.); paola.taviani.arsial@gmail.com (P.T.)

**Keywords:** *Zea mays* L., landraces, germplasm conservation, ddRADseq, SNP

## Abstract

In the framework of a Collaboration Agreement between CREA and ARSIAL, a morpho-phenological, chemical, and genetic characterization of maize populations native to the Lazio region was carried out. During 2022 and 2023, a set of 50 accessions, belonging both to ARSIAL and CREA maize collections, were multiplied in Bergamo. Morpho-phenological descriptors were recorded in the field: plant height, ear height, and male and female flowering time. The grain chemical composition in terms of protein, lipid, starch, ash and fiber was evaluated by near-infrared spectroscopy (NIRS). A double-digest restriction-site-associated DNA sequencing (ddRADseq) strategy was used to genotype the landraces. The two collections were not significantly different in terms of grain chemical composition. On the other hand, the ARSIAL and CREA germplasm showed a different distribution in the three cluster-based population structure obtained by ddRADseq, which largely corresponded to the distribution map of their collection sites. The materials from the Lazio region maintained by ARSIAL and CREA were revealed to be different. The comparison between the two groups of landraces showed the importance of characterizing germplasm collections to promote the recovery and valorization of local biodiversity.

## 1. Introduction

Maize (*Zea mays* L.) is one of the most important cereal crops in human and animal diets worldwide [1]. Aside from providing nutrients for humans and animals, maize serves as a basic raw material in the production of starch, oil, protein, alcoholic beverages, food sweeteners, and fuel.

Open-pollinated populations (i.e., landraces) are genetic materials that have been cultivated by farming communities at a local level and represent a reservoir of valuable alleles that can be exploited for different aims. Landraces are characterized by specific adaptations to the environmental conditions of the cultivation area (tolerant to the biotic and the abiotic stresses of that area) and are closely associated with traditional farming strategies, knowledge, habits, and dialects, and celebrations of the people who developed and continue to grow them [2].

The depletion of genetic resources in food crops was discussed as a public issue in 1992 during the Convention on Biological Diversity [3]. Since then, scientific research has focused on aiding the recovery and maintenance of agrobiodiversity, recognized as a key endeavor to support the sustainability of traditional and modern agricultural systems and food safety and security. For this purpose, genebanks (ex situ conservation) have acquired a key role as a source of genetic resources. Genebanks store representative germplasm samples of crop species and provide access to these materials for their evaluation and integration into modern breeding programs and for conservation purposes. On the other hand, in situ conservation, i.e., cultivating and propagating crops in farmers’ fields, complements genebanks in maintaining local intra- and inter-species agrobiodiversity. A study established to compare in situ and ex situ conservation over 50 years in Mexico [4] indicated that farmers could maintain the genetic diversity of their landraces, and ex situ accessions from genebanks were still representative of the diversity present in farmers’ fields.

The Maize Genebank of CREA—Centre for Cereal and Industrial Crops—in Bergamo maintains the largest Italian ex situ maize collection, with more than 4000 accessions. Among them, maize landraces (720 are Italian and more than 500 are from other countries), which, in the past, represented a source of survival for country people, represent a highly interesting group. They were replaced in the 1950s by hybrids introduced into Italy from the USA, characterized by higher yield and resistance to pathogens. Thanks to an initiative of the former Maize Experimental Station (Stazione Sperimentale per la Maiscoltura) of Bergamo, landraces were collected from Italian regions, stored in Bergamo, and periodically reproduced to conserve seed viability [5].

In the Lazio region, Central Italy, census, morpho-physiological, and genetic characterization of herbaceous germplasms cultivated by local farmers are carried out by ARSIAL (Regional Agency for Agricultural Development and Innovation of Lazio) according to CBD, with the application of Regional Law No. 15 (1 March 2000) for the “Protection of genetic resources of agricultural interest, indigenous in Lazio and at risk of erosion” [6]. This characterization concerns landraces that originated in Lazio territory, that were introduced and integrated into the agroecosystem of Lazio at least 50 years ago, or that disappeared from the region and were collected for public or private genebanks of other regions or countries. As set forth in Article 8 of CBD, the heritage of agricultural genetic resources indigenous to Lazio belongs to the local communities that have preserved them to date. Since 2007, ARSIAL has collected about thirty maize accessions of landraces by Lazio local farmers, conserved ex situ in a regional genebank [7].

A deep characterization is required to aid the monitoring, conservation, and exploitation of agrobiodiversity. Both morphological and molecular methods are employed in estimating genetic diversity in germplasm collections. Although morphological evaluation is time-consuming, requires a large population size, and does not cover the entire genome, it represents an effective approach to identifying phenotypic variation. On the other hand, it can be limited by the effects of the environment on trait expression, and, for this reason, this characterization should be combined with molecular approaches, which play an important role in identifying diverse germplasms because of their high precision and accuracy. Several studies on germplasm characterization in recent years have focused on local Italian varieties [8,9,10,11,12,13,14,15].

High-throughput next-generation sequencing (NGS)-based genotyping technologies offer the unique advantage of generating a large panel of single-nucleotide polymorphism (SNP) markers. Indeed, these technologies are starting to represent the best approach to assessing genetic diversity at different population levels and to de novo SNP discovery, both in model and non-model species, due to their scalability and the possibility to combine different enzymes to digest genomic DNA depending on the plant source and the aims of the research [16,17,18,19].

From this perspective, within an ARSIAL-CREA collaboration, a total of 50 landraces from Lazio were chosen from both genebanks and characterized for morpho-phenological traits in two growing seasons, and, for genetic analysis, a reduced genome representation strategy, with the use of ddRADseq to genotype at low coverage, was applied in the present study.

## 2. Results and Discussion

### 2.1. Morpho-Phenological Logical Parameters

**Figure 1:**
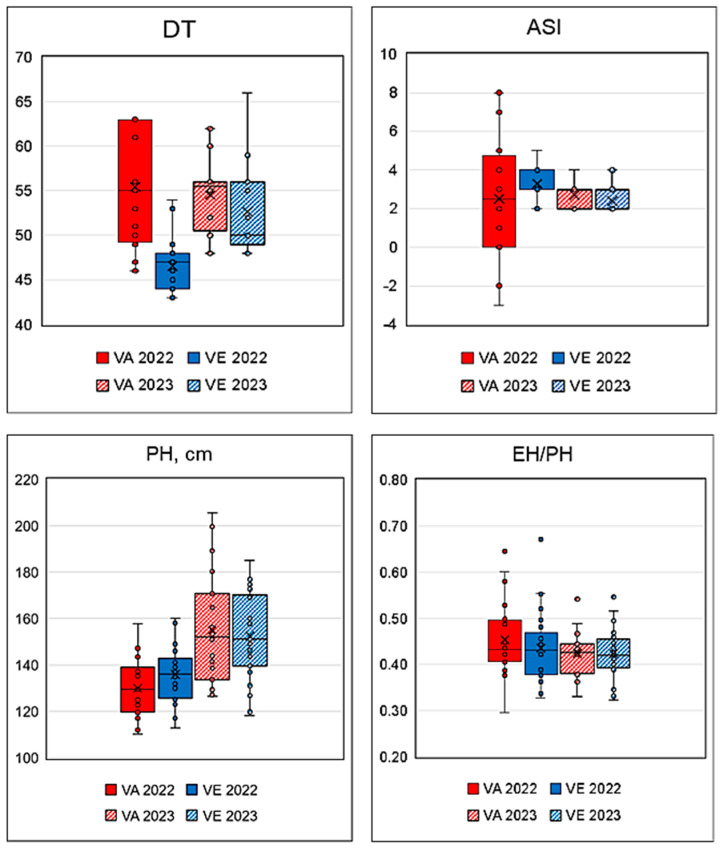
Box-plots of the field traits (DT, days to tasseling; ASI, anthesis–silking interval; PH, plant height; EH/PH, ear height/plant height ratio) registered during 2022 and 2023.

Figure 1 shows the field parameters registered in 2022 and 2023 for VA and VE accessions: days to tasseling (DT), anthesis–silking interval (ASI), plant height (PH), and ear height/plant height ratio (EH/PH). The peculiar climatic conditions in 2022, extremely dry and hot, influenced plant flowering. The mean values for days to tasseling (DT) were compared by t-test and found to be significantly higher (*p* ≤ 0.05) in VA accessions than the values registered for them in 2023 and the VE values. ASI in VA accessions in 2022 had a larger standard deviation than VE, but the mean values of the two groups were similar and did not differ significantly from the data registered in 2023. The mean values for plant height (PH) in 2023 were significantly higher than in 2022 (*p* ≤ 0.01) for both VA and VE, due to better growing conditions and the larger availability of water; the EH/PH ratio remained similar in the two seasons.

### 2.2. Grain Chemical Composition

During multiplication, accessions were revealed to be sensitive to environmental conditions: in particular, in 2022, tassel fertility was strongly reduced by high temperatures and drought, and it was only possible to obtain enough seeds to evaluate the grain chemical composition from half of the harvested accessions. During 2023, eight accessions (VA 383, VE-0220, VE-0320, VE-0369, VE-0528, VE-0531, VE-0568, and VE-0765) again were almost sterile; therefore, the chemical data reported in the present study are related to the 42 landraces successfully multiplied in 2023 (19 VA and 23 VE).

The mean values for the 1000-seed weight and grain chemical compounds for the two groups (VA and VE), as determined by NIRS (Table 1), were compared by t-test and were shown to be not significantly different. The range of variation was wider in VE for all traits except starch. The detailed chemical composition of all accessions is reported in Table S1. ANOVA analysis indicated a significant effect (*p* ≤ 0.01) of the factor genotype on the parameters (Table S2).

A positive and significant correlation (*p* ≤ 0.01) was found between lipid and starch, both negatively correlated to protein content; a negative, significant correlation (*p* ≤ 0.01) was also found between fiber and starch content. All the correlations between seed weight and the other traits were not significant (Table S3).

Grain chemical composition is an important trait in the characterization of traditional germplasm, as it is often used for food production. In industrialized countries, maize is mainly grown for silage and industrial uses. As compared to commercial hybrids, which have high yield and better resistance to environmental stresses, landraces are usually richer in protein and lipid content and show a higher percentage of molecules with a role as antioxidants [20,21,22,23,24]. For this reason, several national and international research programs are now underway, focused on the description and valorization of these genetic materials. Near-infrared (NIR) spectroscopy is a well-established non-destructive screening method used in plant breeding and in the cereal industry for estimating a wide range of chemical components and for the screening of many samples. The reproducibility of the acquired spectra, the number of specific calibrations developed for the main components of plant tissues, and the high prediction performance make it the most efficient method to acquire many chemical data in a reduced span of time [25]. It is therefore frequently applied in quality evaluation.

In a previous study [11], which characterized Italian landraces by NIRS 547, a large variability was observed for grain chemical composition. The variation ranges observed were as follows: 7.91–15.42% dry matter for protein, 2.58–7.74% dry matter for lipid content, and 61.18–67.69% dry matter for starch content. This variability was close to that determined for the 449 landraces originating from other countries, revealing that the technique was not effective in discriminating among the landraces on a geographical basis. An international network (EVA maize) involving European genebanks, research institutes, and breeders is currently studying traditional European landraces, to explore their variability and their possible exploitation as pre-breeding materials [26]. In parallel, a set from these European landraces has been multiplied in Bergamo in recent years and is being characterized for nutritional characteristics [27].

**Figure 2:**
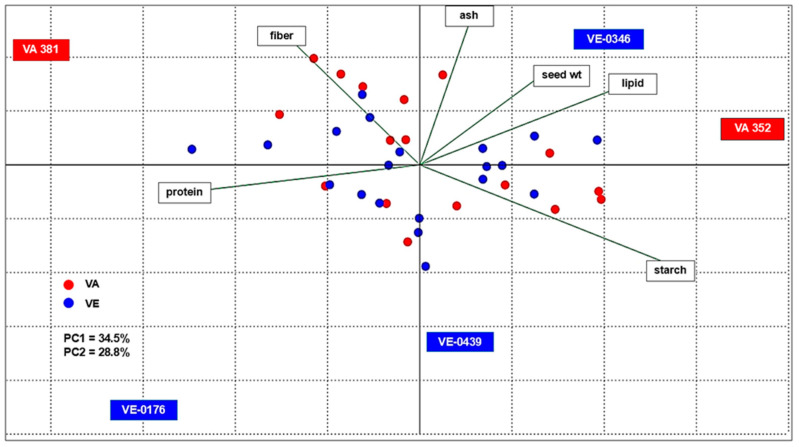
PCA biplot of the grain chemical composition of the landraces analyzed.

To understand how the basic chemical composition characterized the landraces, the results were subjected to a PCA analysis based on a correlation matrix (Figure 2). The first principal component (PC1) accounted for 35.1% of the variability and correlated especially with protein, lipid, and starch. The second principal component (PC2) explained 28.8% of the variability and correlated with the ash and fiber content. VA (red dot) and VE (blue dot) accessions are evenly distributed across the diagram. The accessions that appear to be distinguished from the others are highlighted in the PCA. Two landraces from CREA, VA 381 and VA 352, had opposite compositions, with the former being the richest in protein (13.61%) and fiber (3.44%) and the latter showing the highest starch (68.20%) and lipid (5.79%) content. Among ARSIAL accessions, VE-0346 had high values for 1000-seed weight (270.0 g) and lipid (5.39%) and fiber (3.15%) content. VE-0176 was high in protein (13%) but the lowest in lipid content (3.53%). Finally, VE-0439 had a good starch content (66.74%), but its values for the other traits were lower than the average (Table S1).

### 2.3. Genotyping and SNP Calling

Despite the increasing number of studies on the use of ddRADseq in model [28,29] and non-model crop genotyping [30,31], as well as in the general characterization of the genetic diversity of different taxa [32], so far, its application in maize is still limited. This approach applied addRADseq to genotype the collection of 50 landraces. Overall, as shown in Table 2, after quality-checking the raw data, all the samples preserved >91% of high-quality reads after trimming and >96% of uniquely mapped reads, with a very low percentages of multi- and unmapped reads.

After alignment cleanup, the haplotype-based variant detector FreeBayes was employed to identify genetic variation within each sample’s mapped genome. The minimum coverage, the minimum genotype quality, and the minimum base quality thresholds to call a variant were set to 6, 30, and 30, respectively, and the overall statistics of genetic variants are reported in Tables S4 and S5.

The low-quality variants with a minor allele frequency (MAF) lower than 5%, a quality score below 30, and a read depth below 6, as well as individuals with missing genotype data for more than 90% of the variants, were filtered out using VCFtools. Moreover, InDels were also removed and not considered for downstream population genetic analyses. As summarized in Table S4, after filtering, all the individuals (50) and a total of 15,166 SNPs markers were retained; most samples exhibited less than 10% (0.10) of missing loci, while samples VE-0219 and VE-0346 had higher rates, reaching 15% and 13%, respectively (Figure S1).

The annotation and functional impact evaluation of the genetic variants from the filtered loci was carried out with the VEP (Variant Effect Predictor) software (version v110). All the variants were classified as SNPs, since InDels had been removed earlier from the original VCF file, and the results with the most severe consequences and within coding sequences are reported in Table S4. Overall, the number of SNP loci was uniformly distributed across the 10 maize chromosomes (), with chromosomes 1 and 2 showing the highest proportion of identified variants according to their lengths (Figure S2) [33,34,35].

### 2.4. Population Structure and Germplasm Diversity

**Figure 3:**
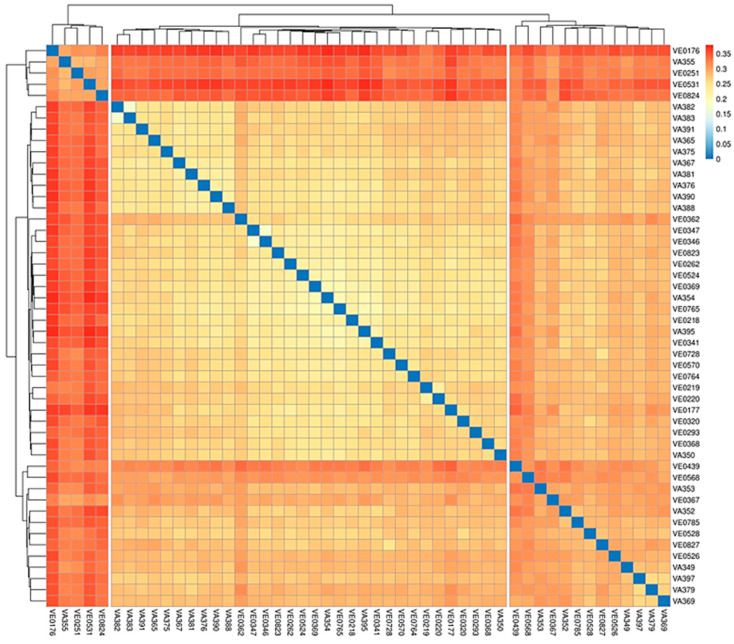
Heatmap showing the p-distance matrix for the 50 genotypes. The heatmap plot depicts the discrepancy of each sample by color intensity. The higher the value, the closer to red, and thus the larger is the discrepancy between two samples. The dendrograms on the top and on the left indicate the genetic relatedness between samples according to the p-distance matrix.

To gain an insight into the population structure and genetic diversity within the maize collection including VA and VE accessions from the Lazio region, several population genetic analyses were conducted. Based on the 15,166 filtered loci, a p-distance matrix, representing the genetic similarity between pairs of samples, was generated. A heatmap showing the similarity across the entire dataset (Figure 3) reveals three distinct clusters within the analyzed germplasm collection.

**Figure 4:**
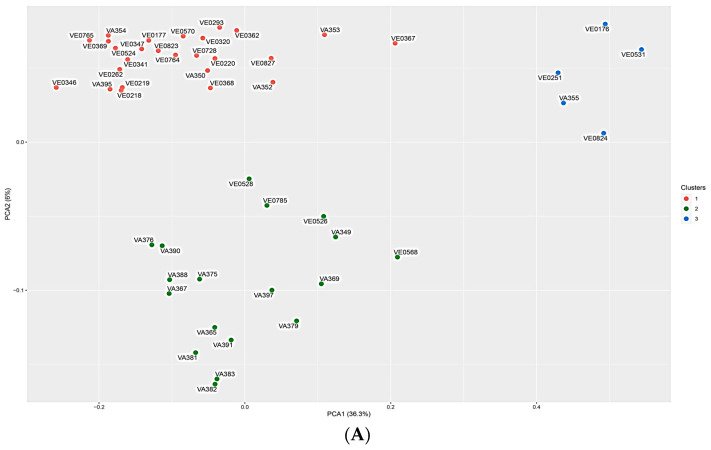

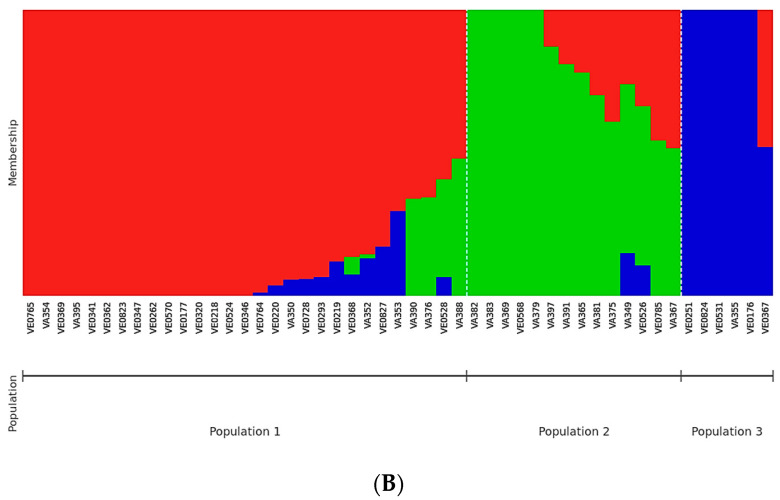
Unveiling the genetic diversity of maize landraces from the Lazio region using ddRADseq. (**A**) PCA based on the Prevosti distance of the similarity between the 50 samples. The three clusters are represented by different colors, with cluster 1 (red), cluster 2 (green), and cluster 3 (blue) grouping 26, 15, and 5 individuals, respectively. The outlier sample VE-0439 was removed. (**B**) Population structure inferred by STRUCTURE. A Distruct plot representing the admixture of populations with the number of expected populations set to 3 (K = 3) is shown. Each landrace is represented on the *X*-axis and visualized into K colors according to its membership coefficient.

To further investigate relatedness within the germplasm collection, the genetic distance between samples was calculated based on the Prevosti distance. Indeed, while the p-distance considers the overall differences in genetic variants, the Prevosti distance focuses on shared alleles at specific loci. Based on the calculated Prevosti distance values, an automated clustering analysis on the top 50 PCs (principal components) was conducted (Figure 4A). STRUCTURE v 2.3.4 [36] was used to study the population structure and genetic relations among the 50 maize landraces from the Lazio region. The BIC (Bayesian information criterion) values were used to estimate the optimal number of clusters that define the population structure. Based on the BIC value plot (Figure S3), samples were grouped into three main clusters in agreement with the PCA. Sample VE-0439, being an outlier, was further removed from all downstream analyses and PCA plots (Figure 4A) where PC1 explained the higher proportion of total variability (36.3%) according to previous results. Clusters 1, 2, and 3 grouped 26, 18, and 5 individuals, respectively. Interestingly, genotypes from ARSIAL (VE) and CREA (VA) collections are mainly separated into cluster 1 and 2, respectively. The three accessions belonging to the “Mais Agostinella” landrace (VE-0218, VE-0220 and VE-0341) were found to be grouped in the same cluster (red), reflecting their origin (Figure 4A). Mais Agostinella is the only landrace registered in the Regional Voluntary Register (last accessed on 16 July 2024) and is maintained in situ/on farms by Vallepietra farmers in the mountains of Valle dell’Aniene (Rome). Moreover, the accession Bufaletta (VE-0219) was also found to be grouped with the Agostinella landrace. Since it was collected from the same location as VE-0218 and VE-0220 (Vallepietra), it is probable that the real genetic origin of Bufaletta is the same as that of Agostinella. Together, the genetic and chemical composition data represent a valuable procedure for identifying landraces in the Lazio region, thus aiding on the journey to their preservation and management at the local community level. Indeed, many autochthonous genetic resources are conserved by farmers and also by participatory methodology; it is therefore important that specific systems are defined for their management. The population structure was separately evaluated without the outlier sample VE-0439 as an input, and the resulting Distruct plot is shown in Figure 4B. Briefly, it assigns individuals to different genetic clusters based on their genetic variation and provides insight into the admixture proportion within a population. The number of expected populations was set to 3 (K parameter) in accordance with the Bayesian clustering results.

Basic population genetic analysis was computed on the STRUCTURE results, and the genetic diversity index was calculated to provide insights into genetic diversity within each of the three clusters. The three populations were genetically different at a significant level based on the *p*-value equal to zero and had similar expected heterozygosity (He) with a range of 0.28–0.31, although population 3 was slightly higher than population 1 and 2, suggesting that it was more diverse at the genetic level than the others. 

Finally, an SNP panel was obtained describing genetic variability within the population; it could represent a valuable genetic resource for further investigations into these accessions.

### 2.5. Genetic/Geographical Association of CREA and ARSIAL Maize Collections

In the 1950s, the former Maize Experimental Station in Bergamo organized the collection of maize landraces grown by farmers in all Italian regions [5]. Forty-nine samples were obtained from Lazio, most of which (27) were from Rieti province, with 17 from Latina and 5 from Rome. The group of 20 CREA landraces chosen to be analyzed in the present study reflected this percentage: 12 VA were from Rieti, 6 were from Latina, and 2 were from Rome. On the other hand, the 30 accessions included by ARSIAL covered all the provinces in Lazio: the largest number (14) came from Frosinone, 5 came from Rieti and Rome, 3 came from Latina, and 2 came from Viterbo (Table 3). Among the two collections, several phenological differences were found. As an example, four landraces from the CREA collection (VA 349, VA 352, VA 353, and VA 355), all from Latina province, have white kernels, whereas no accessions with white kernels were found among those cataloged by ARSIAL in recent years. This suggests that a loss of biodiversity occurred in that area during the transition from the cultivation of landraces to the introduction of hybrids. On the other hand, within the collection from ARSIAL, several accessions showed a dark red kernel color, a characteristic that is not present in the landraces maintained at CREA.

**Figure 5:**
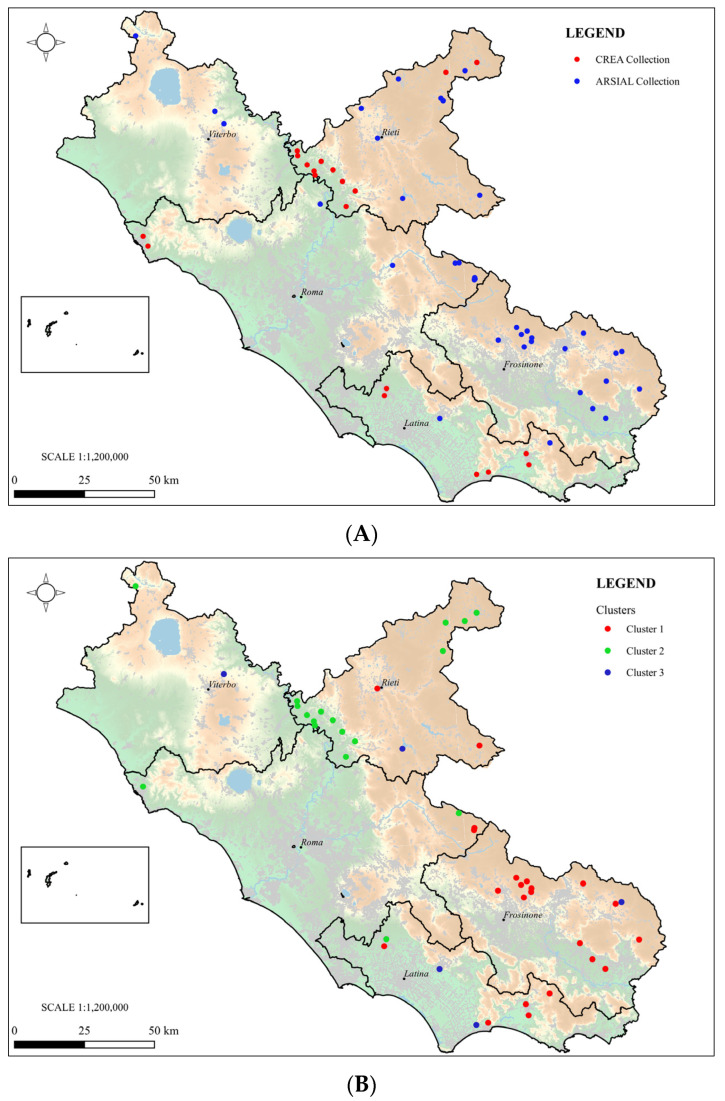
(**A**) Sites of collection of maize landraces in Lazio (red dots, CREA collection; blue dots, ARSIAL collection); (**B**) geographical distribution according to the genetic clusters (cluster1, red; cluster 2, green; cluster 3, blue).

The maps in Figure 5 compare the sites of collection according to GPS coordinates (A) and the geographical distribution of landraces according to the clusters obtained by sequencing (B). Accession VE-0439 originated from another region and was removed from the map shown in Figure 5B. Genetic clustering corresponded quite well to the geographic origin of the landraces. Cluster 1 (red dots) is mainly concentrated in the Southern part of the region, in Frosinone and Latina provinces; cluster 2 (green dots), on the other hand, can be found in Northern Lazio, in Rieti province. This result suggests a defined relation between landraces’ genetic characteristics and traditional in situ conservation sites. This relation is not applicable to cluster 3 (blue dots), as these landraces are spread throughout the entire region.

## 3. Conclusions

The focus of this research was the characterization of maize germplasm from the Lazio region. Two groups of materials were compared: one was composed of landraces collected from farmers in the 1950s and maintained ex situ at CREA Maize Genebank in Bergamo; the other, managed by ARSIAL, included maize accessions still present and grown in the region.

In recent years, maize landraces have been considered quite an interesting material for pre-breeding programs due to their nutritional properties and their characteristics of resilience to environmental stresses. Several national or regional projects have focused on the maintenance and the evaluation of these materials; therefore, several landraces from the CREA maize collection have been studied, with a special focus on those traditionally grown in Northern Italy [8,9,10,11,12,13,14,15]. Some landraces from other regions were also selected for a larger characterization in collaboration with European partners [26,27].

The CREA-ARSIAL agreement offered the opportunity to carry out a detailed description of the traditional populations from the Lazio region, which had not been studied before. The results obtained in the present study, especially from the genetic analyses, indicate that the materials maintained by ARSIAL are mainly different from the germplasm conserved in Bergamo. It can be suggested that the landraces collected from Lazio in the 1950s did not represent all the genetic biodiversity present in the region at that time or that some materials were lost in the following years.

The comparison between the two groups of materials highlighted the importance of germplasm characterization in promoting the recovery and valorization of local biodiversity. This point also highlights the important role of ex situ conservation in maintaining the allelic richness of crop species, which can be exploited in future breeding programs to guarantee food security and safety and to ensure a sustainable relationship with the environment.

## 4. Materials and Methods

### 4.1. Plant Material

**Figure 6:**
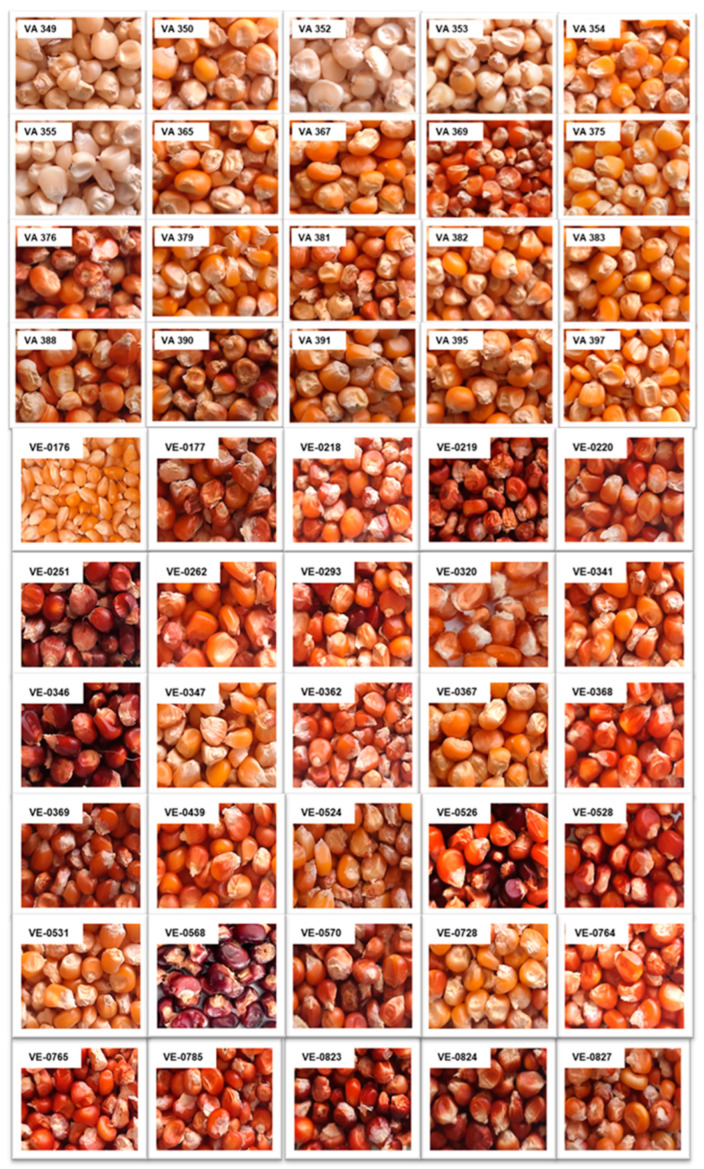
Grains of the maize accessions from the CREA and ARSIAL collections (with codes VA and VE, respectively).

Twenty maize landraces collected in Lazio in 1954 and conserved in the CREA maize genebank (code, VA) and 30 maize accessions collected by ARSIAL in Lazio in recent years (code, VE) were considered in this study (Table 3 and Figure 6).

### 4.2. Plant Multiplication

Plants of each landrace were sown in 2022 (May 12) and 2023 (May 15) on CREA experimental Farm “La Salvagna” (Bergamo, 45°41′42″ N, 9°40′12″ E) in eight plots (4 m long, 20 plants/plot) and were multiplied by controlled pollination in a dedicated field sector (nursery). Fertilizer (kg ha^−1^: N = 280, P_2_O_5_ = 115, K_2_O = 120) and irrigation were applied during the growing season to limit drought stress. The method of controlled pollination is used for the multiplication of genebank accessions; to prevent contamination by pollen migration from outside the multiplication plots, silks are covered with paper envelopes and tassels with pollen bags, followed by swift and accurate pollination.

### 4.3. Weather Conditions

**Figure 7:**
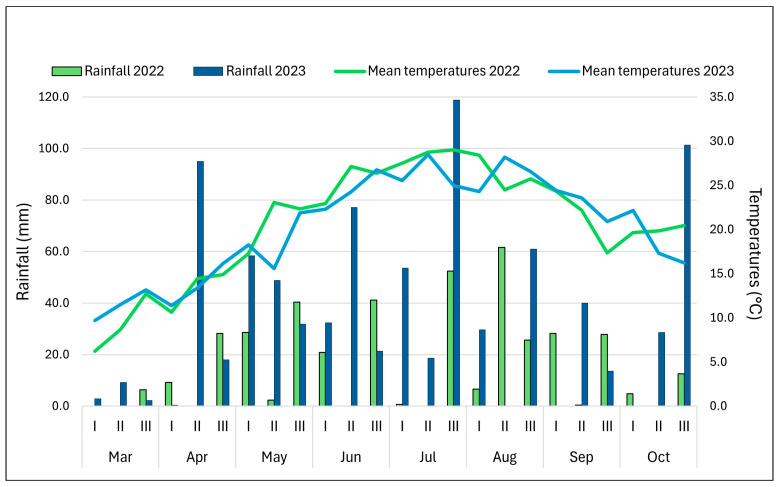
Weather conditions during 2022 and 2023 in Bergamo: rainfall (mm) and temperatures (°C).

The graph in Figure 7 shows the weather conditions in Bergamo (rainfall, colored bars, mm; temperatures, colored lines, °C) during 2022 and 2023. The two years showed different climate trends; during 2022, a very low amount of precipitation was recorded compared to 2023, when the rainfall was very high, especially at the end of April, for the entire month of May, and in July. Temperatures were higher in 2022 than in 2023 from May through the first ten days in August. On the contrary, in 2023, temperatures were higher than in 2022 from the 10th August to the 10th October. The combination of low rainfall and high temperature during flowering time in 2022 determined a high level of stress on plants, which led to major problems with seed production.

### 4.4. Field Traits

During the agronomical season, phenological and morphological maize field traits were evaluated following published descriptors [37], as detailed below.

Days to tasseling (anthesis, male flowering); DT; IPGRI descriptor, 4.1.1: the number of days from sowing to when 50% of the plants have shed pollen. Days to silking (female flowering); DS; IPGRI descriptor, 4.1.2: the number of days from sowing to when silks have emerged on 50% of the plants. Plant height; PH; IPGRI descriptor, 4.1.4: the distance from ground level to the base of the tassel after the milk stage, measured in cm, with the observed value recorded for an average of 10 plants per plot. Ear height; EH; IPGRI descriptor, 4.1.5: the distance from ground level to the node bearing the uppermost ear after the milk stage, measured in cm, with the observed value recorded for an average of 10 plants per plot. The EH/PH ratio was calculated using the measurements of 10 plants per plot. These traits were chosen for the preliminary phenotypic description of the landraces because they provide key information related to adaptation in different environments.

Meteorological data (max temperature, °C; rainfall, mm) were registered in the period from March to October each year.

### 4.5. Grain Characterization

At maturity, the materials derived from controlled pollination were manually harvested; ears were dried at 40 °C up to 14% relative humidity and then were shelled, and the 1000-grain weight was recorded and averaged over three measurements.

An aliquot of each sample was ground with a Retsch ZM 200 lab mill (sieve: 0.5 mm) and kept at 7 °C until analysis. Samples were then analyzed by near-infrared spectroscopy (NIRS) using a Spectra-Star XT-R spectrometer (Unity Scientific, Westborough, MA, USA) in the range 680–2600 nm. Protein, lipid, starch, fiber, and ash contents were determined using a specific calibration curve and expressed as a percentage on a dry matter basis. Each value is the average of three replicates.

### 4.6. Statistical Analysis

The mean and standard deviation of the chemical data were calculated by Excel^®^. The effect of genotypes on the chemical composition was analyzed by a one-way factorial analysis of variance (ANOVA) using the open-source software R 4.4.2. [38]. Correlation analysis, a t-test, and multivariate analysis (principal component analysis, PCA) were carried out using the software PAST version 2.12 according to Hammer et al. [39].

### 4.7. DNA Extraction, Library Preparation, and Sequencing

Leaves from 50 maize accessions were collected on June 29, 2022, immediately frozen in dry ice, and stored at −80 °C until being processed. Genomic DNA was extracted from maize leaves using the ReliaPrep™ gDNA Tissue Miniprep System following the maize-specific protocol provided with the technical information (Promega Italia S.R.L., Milan, Italy).

Double-digest RAD sequencing (ddRADseq) [40,41,42] is a reduced-representation sequencing (RRS) method that, by means of double enzymatic digestion, reduces the whole-genome complexity in order to sequence the genomic fragments associated with restriction enzyme cut sites. The ddRAD library preparation and sequencing were performed by IGA technology services S.R.L. (Udine, Italy) using an IGATech custom protocol, with minor modifications with respect to Peterson’s double-digest restriction-site-associated DNA preparation [39]. The resulting libraries are checked with both a Qubit 2.0 Fluorometer (Invitrogen, Carlsbad, CA, USA) and Bioanalyzer DNA assay (Agilent technologies, Santa Clara, CA, USA). Libraries were sequenced with 150 cycles in paired-end mode using a NovaSeq 6000 instrument following the manufacturer’s instructions (Illumina, San Diego, CA, USA).

### 4.8. Alignment, Variant Calling, and Annotation

Bioinformatic analysis to define population structure and genetic diversity between the populations was performed by Mentotech. S.R.L. (Napoli, Italy). Firstly, a quality check on the Illumina raw sequencing data was carried out with the software BBDuk v38.90 [43]. Low-quality portions were removed while preserving the longest high-quality part of NGS reads. The minimum length was set to 35 bp, and the quality score was set to 35. Trimmed reads were mapped against the reference genome of *Zea mays* (assembly Zm-B73-REFERENCE-NAM-5.0; accession number, GCA_902167145.1), employing the Minimap2 alignment tool [44] with default parameters. Overall, all the samples preserved above 91% of reads after trimming and above 96% of uniquely mapped reads, with very low percentages of multi- and unmapped reads.

The FreeBayes v1.3.7 tool [45] was used to identify genetic variations such as single-nucleotide polymorphisms (SNPs) within each sample’s mapped genome. The minimum coverage, the minimum genotype quality, and the minimum base quality thresholds to call a variant were set to 6, 30, and 30, respectively. Additional quality control (QC) checks on the obtained VCF file were conducted using VCFtools v0.1.16 [46] to discard variants with a minor allele frequency (MAF) lower than 5%, a quality score below 30, or a read depth below 6, as well as individuals with missing genotype data for more than 90% of the variants. InDels variants (insertions and deletions) were also removed and not considered for downstream population genetic analyses. The software VCFtools (version v0.1.16) was also used to identify samples with a high rate of missing data.

The annotation and functional impact evaluation of the genetic variants from the filtered VCF was carried out with the VEP v110 (Variant Effect Predictor) software [47].

### 4.9. Population Structure and Genetic Diversity

A p-distance matrix, representing the genetic similarity between pairs of samples, was generated from the filtered VCF file using the tool VCF2Dis v1.50 (https://github.com/BGI-shenzhen/VCF2Dis, accessed on 1 June 2023). The genetic distance between samples was calculated based on the Prevosti distance [48]. Briefly, while the p-distance considers the overall differences in genetic variants, the Prevosti distance focuses on shared alleles at specific loci. Based on the calculated Prevosti distance values, an automated clustering analysis was conducted.

The number of unique alleles present in a population, considering both rare and common alleles, known as the allelic richness measure (AR), was evaluated using the allel.rich function from the PopGenReport R package [49]. Population structure was separately evaluated with a parametric strategy, the fastStructure algorithm [50], using the filtered VCF file without the outlier sample VE-0439 as the input. Briefly, it assigns individuals to different genetic clusters based on their genetic variation and provides insights into the admixture proportion within a population. The number of expected populations was set to 3 (K parameter) in accordance with the clustering results.

## Figures and Tables

**Table 1:** Mean values and range of variation (% dry matter) of the main grain chemical components and 1000-seed weight (g) in the two groups of landraces (VA and VE).

	Protein	Lipid	Fiber	Ash	Starch	1000-Seed Weight
Mean VA ± SD	12.60 ± 0.90	4.84 ± 0.44	2.67 ± 0.32	1.95 ± 0.15	66.01 ± 1.46	269.9 ± 48.0
Range	11.10–13.70	4.18–5.79	2.22–3.44	1.63–2.26	62.37–68.20	163.3–330.7
Mean VE ± SD	12.61 ± 0.69	4.58 ± 0.49	2.55 ± 0.37	1.86 ± 0.30	66.14 ± 0.77	264.0 ± 43.4
Range	10.59–13.90	3.53–5.50	1.35–3.10	0.77–2.24	64.59–67.32	124.0–350.7

**Table 2:** Trimming and mapping statistics before and after running the analyses. The average values of the 50 genotypes are reported.

Reads Before QC	Reads After QC	Conserved Reads	Mapped Reads	% Mapped Reads
527,313.864	499,626.220	94.76	487,132.236	97.43

**Table 3:** List of landraces from CREA and ARSIAL included in this study and sites of collection.

CREA or ARSIAL Code	Name	Site of Collection	Province	Altitude m Above Sea Level
VA 349	Bianco perla	Cisterna	Latina	77
VA 350	Chiaccarino	Pontinia	Latina	4
VA 352	Quarantino	Fondi	Latina	8
VA 353	Granoturco di Pantano	Fondi	Latina	8
VA 354	Quarantino	Terracina	Latina	22
VA 355	Mazzeco	Terracina	Latina	22
VA 365	Nostrano	Poggio Mirteto	Rieti	130
VA 367	Ottofile	Fara in Sabina	Rieti	482
VA 369	Marano nostrano	Farfa-Granica	Rieti	138
VA 375	Primaticcio	Cantalupo in Sabina	Rieti	297
VA 376	Nostrale	Cittareale	Rieti	962
VA 379	Brigantino	Amatrice Patarico	Rieti	955
VA 381	Zeppe	Magliano Sabina	Rieti	222
VA 382	Quarantino	Stimigliano	Rieti	207
VA 383	Nostrale	Stimigliano	Rieti	207
VA 388	Nostrale	S. Polo-Tarano	Rieti	184
VA 390	Nostrale	Collevecchio	Rieti	245
VA 391	Nostrale	Magliano Sabina	Rieti	222
VA 395	Locale o Granone o Turco	Civitavecchia	Roma	10
VA 397	Quarantino	Civitavecchia	Roma	10
VE-0176	Maranino	Settefrati	Frosinone	759
VE-0177	Filesedici	Villa S. Lucia	Frosinone	91
VE-0218	Agostinella	Vallepietra	Roma	799
VE-0219	Bufaletta	Vallepietra	Roma	799
VE-0220	Agostinella	Vallepietra	Roma	748
VE-0251	Vitorchiano Rosso	Vitorchiano	Viterbo	295
VE-0262	Marano	Unknown	Rieti	385
VE-0293	Gratigno	Veroli	Frosinone	765
VE-0320	Rantign	Roccasecca	Frosinone	177
VE-0341	Agostinella	Vallepietra	Roma	750
VE-0346	Rantign rosso	Campodimele	Latina	394
VE-0347	Rantign giallo	Campodimele	Latina	394
VE-0362	Filesedici	Settefrati	Frosinone	487
VE-0367	Granturco	Collepardo	Frosinone	807
VE-0368	Granturco	Veroli	Frosinone	720
VE-0369	Granturco	Veroli	Frosinone	942
VE-0439	Granturco da polenta	Civitella San Paolo	Roma	198
VE-0524	Mais di Alatri	Alatri	Frosinone	465
VE-0526	Mais Amatrice	Amatrice	Rieti	965
VE-0528	Quarantino (Borbona)	Amatrice	Rieti	758
VE-0531	Mais Sezze	Sezze	Latina	17
VE-0568	Mais rosso	Camerata Nuova	Roma	1356
VE-0570	Mais ottofila	Veroli	Frosinone	512
VE-0728	Mais ottofile	Aquino	Frosinone	110
VE-0764	Mais paesano	Veroli	Frosinone	551
VE-0765	Sconosciuto	Borgorose	Rieti	837
VE-0785	Sconosciuto	Proceno	Viterbo	363
VE-0823	Sconosciuto	Campoli Appennino	Frosinone	592
VE-0824	Mais nostrano	Colle di Tora	Rieti	751
VE-0827	Mais ottofile	Vallerotonda	Frosinone	576

## Data Availability

All relevant data are contained within the article or Supplementary Materials.

## Supplementary Materials

The following supporting information can be downloaded at: https://www.mdpi.com/article/10.3390/plants13223249/s1, Figure S1: Plot depicting the proportion of missing loci for each sample; Figure S2: Distribution of genetic variants (SNPs) on chromosomes; Figure S3: Values of BIC versus number of clusters. Table S1: Chemical composition (% dry matter), 1000-seeds weight (g) and kernel type of CREA and ARSIAL accessions multiplied in 2023; Table S2: Variance analysis of grain chemical compounds and 1000-seeds weight of the landraces (DF = 41); Table S3: Correlation analysis (n = 42) among the grain chemical compounds of the landraces; Table S4: Statistics of the number of SNPs and Indels for the entire dataset; Table S5. Number and proportion of samples and loci kept after Quality Check (QC).
