# Supplementary material for: Morpho-Phenological, Chemical, and Genetic Characterization of Italian Maize Landraces from the Lazio Region
Source: Plants (Basel). 2024 Nov 20;13(22):3249. doi: 10.3390/plants13223249 (PMC11598630; doi:10.3390/plants13223249)
Supplement: Supplementary file 1 [file plants-13-03249-s001.zip › plants-3264156-supplementary-final (11.20).pdf]

Redaelli et al.

Morpho-Phenological, Chemical and Genetic Characterization of Italian Maize Landraces from the Lazio Region

Supporting information

TABLES

Table S1. Chemical composition (% dry matter), 1000-seeds weight (g) and kernel type of CREA and ARSIAL accessions multiplied in 2023

CREA / ARSIAL code	protein	lipid	fiber	ash	starch	1000-seeds weight	Kernel type
**VA349**	12.22 ± 0.06	4.97 ± 0.10	2.43 ± 0.16	1.63 ± 0.07	67.37 ± 0.39	303.3 ± 5.0	flint
**VA350**	13.67 ± 0.11	4.94 ± 0.08	2.44 ± 0.13	2.06 ± 0.19	65.94 ± 0.22	302.7 ± 16.4	flint
**VA352**	11.08 ± 0.07	5.79 ± 0.16	2.43 ± 0.18	1.97 ± 0.14	68.20 ± 0.34	323.3 ± 1.2	flint
**VA353**	11.64 ± 0.11	4.85 ± 0.16	2.73 ± 0.03	1.67 ± 0.19	66.70 ± 0.38	262.0 ± 18.0	intermediate
**VA354**	13.21 ± 0.09	4.63 ± 0.27	2.66 ± 0.17	1.91 ± 0.15	65.84 ± 0.72	210.7 ± 8.1	flint
**VA355**	11.98 ± 0.06	5.00 ± 0.29	2.28 ± 0.26	1.81 ± 0.28	67.35 ± 1.14	312.0 ± 6.9	intermediate
**VA365**	12.22 ± 0.13	5.00 ± 0.11	2.81 ± 0.07	2.26 ± 0.20	65.39 ± 0.46	268.7 ± 13.3	flint
**VA367**	13.01 ± 0.11	4.68 ± 0.19	3.11 ± 0.16	2.00 ± 0.39	64.44 ± 0.74	330.7 ± 8.1	flint
**VA369**	13.57 ± 0.22	5.18 ± 0.09	2.22 ± 0.26	1.91 ± 0.05	66.81 ± 1.41	163.4 ± 6.4	flint
**VA375**	13.09 ± 0.05	4.72 ± 0.09	2.33 ± 0.11	2.04 ± 0.26	67.25 ± 0.52	238.7 ± 5.0	flint
**VA376**	12.22 ± 0.01	4.51 ± 0.04	2.85 ± 0.11	1.95 ± 0.10	65.75 ± 0.20	258.7 ± 4.6	flint
**VA379**	12.10 ± 0.19	5.44 ± 0.18	2.44 ± 0.11	1.89 ± 0.16	67.93 ± 0.51	227.3 ± 7.0	flint
**VA381**	13.62 ± 0.07	4.30 ± 0.11	3.44 ± 0.18	2.08 ± 0.16	62.37 ± 0.32	202.7 ± 5.0	flint
**VA382**	12.38 ± 0.13	4.18 ± 0.08	2.40 ± 0.09	1.91 ± 0.10	65.95 ± 0.30	247.3 ± 6.1	flint
**VA388**	12.70 ± 0.01	4.94 ± 0.14	3.00 ± 0.10	1.98 ± 0.04	64.92 ± 0.18	283.3 ± 12.9	flint
**VA390**	12.47 ± 0.13	4.18 ± 0.09	2.87 ± 0.08	2.18 ± 0.07	64.68 ± 0.40	328.7 ± 4.2	semi-flint
**VA391**	12.83 + 0.15	4.91 ± 0.16	2.89 ± 0.17	1.92 ± 0.26	65.46 ± 0.16	320.7 ± 13.3	flint
**VA395**	13.08 ± 0.08	4.29 ± 0.11	2.84 ± 0.18	1.95 ± 0.17	64.51 ± 0.19	302.7 ± 4.6	flint
**VA397**	12.37 ± 0.10	5.44 ± 0.20	2.66 ± 0.23	2.00 ± 0.03	67.34 ± 0.48	240.7 ± 4.6	flint
**VE_0176**	13.00 ± 0.04	3.53 ± 0.19	2.81 ± 0.24	0.77 ± 0.11	66.33 ± 0.73	124.0 ± 8.7	flint
**VE_0177**	13.03 ± 0.12	4.90 ± 0.18	2.69 ± 0.21	2.13 ± 0.12	65.33 ± 0.39	251.3 ± 9.5	flint
**VE_0218**	12.80 ± 0.10	4.54 ± 0.15	2.70 ± 0.14	1.78 ± 0.15	65.48 ± 0.40	230.0 ± 6.9	flint
**VE_0219**	12.67 ± 0.09	4.46 ± 0.07	3.00 ± 0.11	2.23 ± 0.10	65.79 ± 0.28	268.0 ± 5.3	flint
**VE_0251**	12.65 ± 0.11	4.41 ± 0.12	2.36 ± 0.14	1.69 ± 0.22	66.51 ± 0.62	262.7 ± 6.1	flint
**VE_0262**	12.29 ± 0.12	5.50 ± 0.41	2.39 ± 0.17	1.94 ± 0.15	66.82 ± 0.72	312.0 ± 6.9	flint
**VE_0293**	13.90 ± 0.01	4.83 ± 0.19	2.60 ± 0.12	2.01 ± 0.14	64.70 ± 0.13	234.7 ± 1.2	flint
**VE_0341**	13.01 ± 0.23	4.78 ± 0.31	2.44 ± 0.22	1.96 ± 0.44	67.16 ± 0.39	294.7 ± 4.2	flint
**VE_0346**	10.59 ± 0.11	5.39 ± 0.11	3.15 ± 0.31	2.24 ± 0.24	66.41 ± 0.77	270.0 ± 7.2	flint

**VE_0347**	12.50 ± 0.03	5.14 ± 0.06	2.77 ± 0.09	1.80 ± 0.04	66.54 ± 0.44	285.3 ± 5.0	flint
**VE_0362**	12.55 ± 0.09	5.08 ± 0.07	2.60 ± 0.11	1.95 ± 0.16	66.83 ± 0.08	248.0 ± 17.4	flint
**VE_0367**	13.27 ± 0.08	4.53 ± 0.05	2.56 ± 0.24	1.97 ± 0.09	66.28 ± 0.53	290.0 ± 10.6	flint
**VE_0368**	11.73 ± 0.18	4.90 ± 0.14	2.42 ± 0.33	1.81 ± 0.22	66.74 ± 0.43	256.0 ± 8.0	flint
**VE_0439**	12.49 ± 0.24	3.82 ± 0.18	1.35 ± 0.34	1.69 ± 0.17	66.77 ± 0.84	252.0 ± 0.0	flint
**VE_0524**	13.24 ± 0.08	4.00 ± 0.12	3.10 ± 0.09	1.75 ± 0.10	64.59 ± 0.38	263.3 ± 7.0	flint
**VE_0526**	13.42 ± 0.06	4.47 ± 0.33	2.19 ± 0.24	2.21 ± 0.25	66.47 ± 0.46	214.0 ± 3.5	flint
**VE_0570**	12.35 ± 0.07	4.14 ± 0.07	2.69 ± 0.20	1.66 ± 0.15	65.76 ± 0.45	284.0 ± 8.7	flint
**VE_0728**	12.91 ± 0.10	4.64 ± 0.20	2.59 ± 0.11	1.81 ± 0.07	65.62 ± 0.60	319.3 ± 4.2	flint
**VE_0764**	12.52 ± 0.08	4.28 ± 0.04	2.42 ± 0.08	1.75 ± 0.07	66.50 ± 0.11	275.3 ± 6.1	flint
**VE_0785**	12.41 ± 0.14	4.56 ± 0.14	2.72 ± 0.06	2.01 ± 0.04	64.71 ± 0.22	240.0 ± 12.2	flint
**VE_0823**	12.30 ± 0.06	4.64 ± 0.11	2.44 ± 0.12	1.76 ± 0.10	66.33 ± 0.46	350.7 ± 13.3	flint
**VE_0824**	11.49 ± 0.09	4.84 ± 0.17	2.50 ± 0.08	2.15 ± 0.13	66.32 ± 0.18	257.3 ± 10.1	flint
**VE_0827**	12.97 ± 0.18	3.94 ± 0.16	2.14 ± 0.32	1.75 ± 0.24	67.32 ± 0.44	288.7 ± 13.3	flint

Table S2. Variance analysis of grain chemical compounds and 1000-seeds weight of the landraces (DF = 41)

Factor	Mean Square	F
Protein	1.3811	85.40 **
Lipid	0.6719	19.22 **
Fiber	0.3577	10.18 **
Ash	0.1837	6.04 **
Starch	3.7739	13.65 **
1000-seeds weight	6,100.12	78.96 **

Table S3. Correlation analysis (n = 42) among the grain chemical compounds of the landraces

	Protein	Lipid	Fibre	Ash
Lipid	-0.40 ***			
Fibre	ns	ns		
Ash	ns	0.43***	ns	
Starch	-0.44 ***	0.40***	-0.65***	ns

Table S4. Statistics of the number of SNPs and Indels for the entire dataset

Identified SNPs and InDels	Number of genetic variants in the entire dataset
SNPs	846,917
singleton SNPs	51,823
Indels	136,637
singleton Indels	6,190
SNP/Indel ratio	6.20
Insertion/Deletion ratio	0.93
Transitions loci (Ts)	532,755

Transversions loci (Tv)	307,414
Ts/Tv ratio	1.73

Table S5. Number and proportion of samples and loci kept after Quality Check (QC).

**Parameters**	**Before_QC**	**After_QC**	**Kept_percentage**
Number of samples	50	50	100 %
Number of loci	1,091.062	15,166	1.4 %

FIGURES

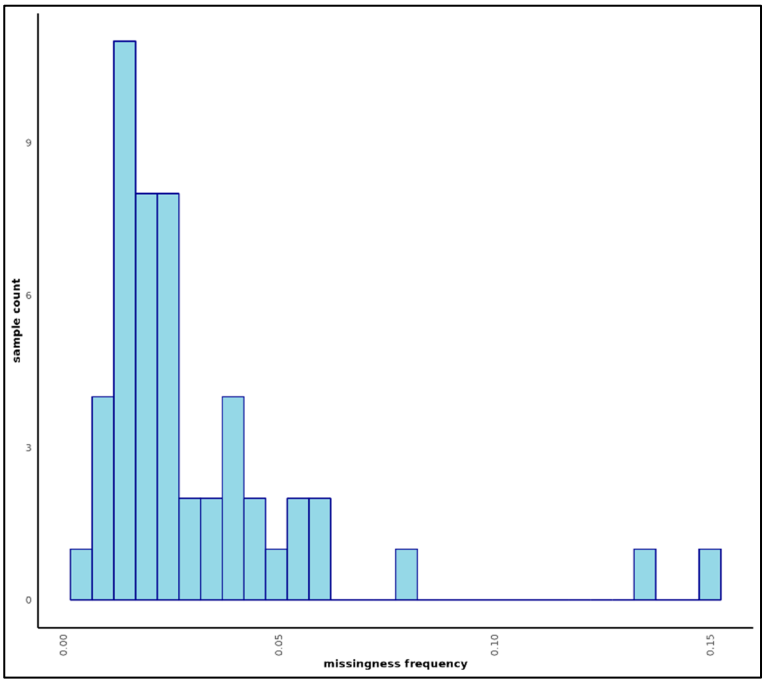

Figure S1. Plot depicting the proportion of missing loci for each sample.

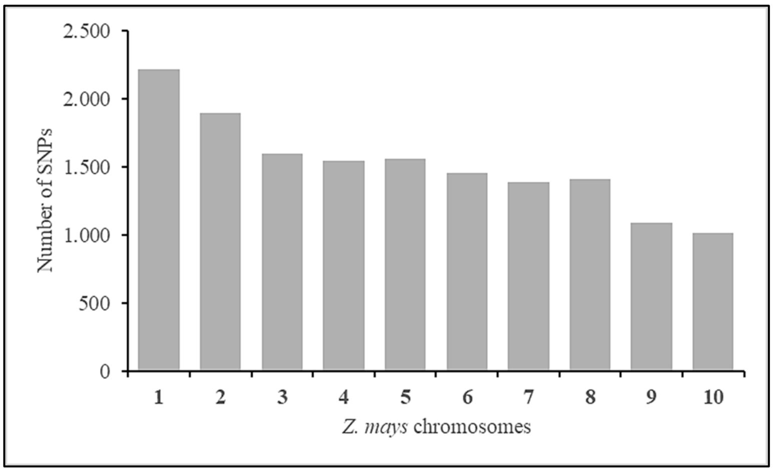

Figure S2. Distribution of genetic variants (SNPs) on chromosomes.

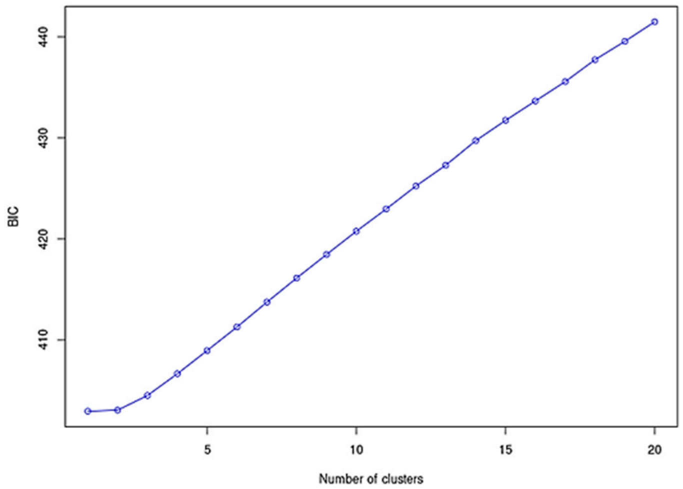

Figure S3. Values of BIC versus number of clusters. BIC (Bayesian information criterion) values on Y-axis were plotted against different numbers of clusters.
